# Isolation of Bioactive Compounds (Carotenoids, Tocopherols, and Tocotrienols) from *Calendula Officinalis* L., and Their Interaction with Proteins and Oils in Nanoemulsion Formulation

**DOI:** 10.3390/molecules29174184

**Published:** 2024-09-04

**Authors:** Kamil Haładyn, Aneta Wojdyło, Paulina Nowicka

**Affiliations:** Department of Fruit, Vegetable and Plant Nutraceutical Technology, Faculty of Biotechnology and Food Science, Wrocław University of Environmental and Life Sciences, 51-630 Wrocław, Poland; kamil.haladyn@upwr.edu.pl (K.H.); aneta.wojdylo@upwr.edu.pl (A.W.)

**Keywords:** emulsion, pot marigold, protein, bioactive compounds, antioxidant activity, ability to inhibit α-amylase and α-glucosidase

## Abstract

*Calendula officinalis* L. has numerous health-promoting properties due to the presence of a large number of lipophilic compounds. Their effective delivery to the body requires the use of an appropriate technique such as emulsification. So, the main purpose of this study was to understand how the profile of lipophilic compounds from pot marigold and the pro-health potential are shaped by different types of protein, oil, and drying techniques in o/w nanoemulsion. To obtain this, the profiles of carotenoid compounds and tocols were measured. Additionally, antioxidant potential and the ability to inhibit α-amylase and α-glucosidase were measured. Pea protein emulsion exhibited a higher final content of carotenoid compounds (23.72–39.74 mg/100 g), whereas those with whey protein had stronger α-amylase inhibition (487.70 mg/mL). The predominant compounds in the studied nanoemulsions were β-carotene (between 19% and 40%), followed by α-tocopherol/γ-tocopherol. The type of proteins shaped the health-promoting properties and determined the content of health-promoting compounds.

## 1. Introduction

*Calendula officinalis* L., commonly referred to as “pot marigold” or “common marigold”, belongs to the Asteraceae family and traces its origins to the Mediterranean region. The inflorescence of the marigold is characterized by its orange or yellow color [1]. Extracts from *Calendula officinalis* L. are acknowledged for numerous biological activities [2]. The wide range of therapeutic attributes of pot marigold—including its antioxidant, antimicrobial [3], antibacterial, antifungal, antiviral and anti-inflammatory [4], antidiabetes, anti-cancer, and hepatoprotective attributes—can be ascribed mainly to its rich composition of bioactive substances. Notably, these include carotenoids, polyphenolic compounds, terpenoids, triterpeneol esters, triterpenoids, saponins, sterols, flavonoids, essential oils, amino acids, and tocopherols [5].

Due to their orange color, marigold flowers are characterized by a high presence of carotenoids. Carotenoid compounds are natural pigments present in plants and are responsible for the yellow, orange, or red colors in various plants [6,7,8]. These compounds exhibit numerous preventive properties, in the context of counteracting the onset of cardiovascular diseases, obesity, and inflammation. Owing to their antioxidant properties, carotenoids possess the ability to capture free radicals, protecting biological membranes from oxidative damage. In addition, some carotenoids are recognized for their provitamin A status [6,7,9].

However, the limited bioavailability of these bioactive compounds curtails the industrial application of carotenoids [6]. Additionally, their intrinsic structure renders carotenoids susceptible to degradation, leading to discoloration and reduced biological activity [10].

Employing emulsions as carriers for lipids presents a promising avenue for effectively delivering lipophilic health-promoting substances to the body. Such techniques facilitate the enzymatic digestion of the formed complexes in the small intestine, promoting easier dissolution and absorption of carotenoids. Moreover, decreasing the size of lipid droplets within an emulsion system can increase the bioavailability of the infused compounds [11]. An emulsion characterized by a particle size of less than 500 nm is termed a nanoemulsion [12,13]. These are extensively used across diverse sectors, including food production, pharmaceuticals, cosmetics, and healthcare [13]. Primarily, nanoemulsions are used to increase the bioavailability of bioactive substances, which amplifies the absorption of encapsulated compounds within the body [12]. In addition, this technique is aimed to protect compounds in the gastrointestinal tract from earlier degradation [14].

For nanoemulsion preparation, two types of surfactants are used. Given the present consumer demand for exclusively natural food products, natural emulsifying agents, e.g., plant and animal proteins, are replacing synthetic emulsifiers [12,15]. Various emulsifiers can create emulsion systems with different physicochemical properties. However, individual emulsion components may have different effects on its final properties. The use of different types of protein may potentially influence the development of specific health-promoting properties of the obtained material, but also affect the profile of the introduced bioactive compounds. This may be of key importance in the context of designing food with the desired properties and is not yet validated in complex matrices of bioactive compounds, including those isolated from plant raw materials. To do this, it is necessary to first know the influence of the individual components of the emulsion and the method of its preparation on the development of functional properties.

Therefore, for this study, we aimed to evaluate the effects of using specific plant proteins (namely pea protein isolate, soy protein isolate, and chia protein) and animal proteins (such as whey protein isolate and whey protein) in combination with different drying methods and oil types in the formation of oil in water (o/w) nanoemulsions infused with carotenoid compounds derived from marigold flowers. For this purpose, we determined the in vitro biological activity of the obtained nanostructures, accompanied by a comprehensive analysis of the profile of lipophilic bioactive compounds (carotenoids, tocopherols, and tocotrienols). To our knowledge, there was no prior research centered on formulating nanoemulsions that incorporate complexes of carotenoid compounds extracted from the common marigold flower. Available research focuses only on the use of individual carotenoid compounds, mainly standards, and the study of their activity in the obtained nanostructure, and not on the entire complex of compounds. Moreover, no comparison of the impact of individual proteins, type of oil, and drying method on the health-promoting activity of nanoemulsions based on phytochemical extract from marigold and the profile of bioactive compounds as a result of these processes was conducted so far.

## 2. Results and Discussion

### 2.1. Particle Size and PDI

One of the most important parameters characterizing nanoemulsions is particle size distribution. Table 1 presents the particle size and PDI results for emulsions enhanced with 3% (*m*/*v*) of isolated carotenoid compounds in oil. The kind of oil and emulsifier used significantly influenced the measurement outcomes. Emulsions containing sunflower oil exhibited a particle size distribution ranging approximately between 194 and 4598 nm, while those with hemp oil varied between 198 and 6799 nm. The smallest values, for both oil types, were noted in formulas infused with whey protein (194 nm and 199 nm). There was not a statistically significant difference in particle size for the nanoemulsion with sunflower oil when considering the variant with whey protein isolates versus whey protein (197 nm and 199 nm, respectively). Whey protein isolate, enriched with globular proteins such as β-lactoglobulin and α-lactoglobulin, is an optimal choice for crafting nanoemulsions. Owing to its structure, it possesses the capability to adsorb at the oil–water interface, thereby forming a cohesive aggregate typical of a nanoemulsion [16]. Among the emulsions made of hemp oil and plant proteins, there was no significant difference between soy protein isolate and pea protein isolate (257 nm and 265 nm, respectively). Conversely, Jarzębski et al. [17] obtained a lower particle size result (209 nm) in a nanoemulsion containing hemp seed oil, lecithin (5%), and pea protein (0.4%) compared to the variant described above. The study showed that the addition of pea protein contributed to a lower particle size of the emulsion compared to an emulsion composed of a single emulsifier—lecithin.

Though proteins are touted as potential emulsifiers, not every material is technologically apt for nanoemulsion generation. Soy protein isolate can be used in nanoemulsion production only if there is either a high concentration of it (>1%) or a low concentration of oil (<0.1%) while applying high-energy techniques [18]. Nanoemulsions formulated with peanut oil and soy protein isolate and subjected to high-pressure homogenization have particle sizes ranging roughly between 120 and 740 nm. This size variance depends on the protein concentration: the higher the protein concentration, the smaller the emulsion particle size. Such an occurrence is likely attributed to the enhanced encapsulation of oil droplets by the protein, preventing droplet aggregation [19]. The largest particle size was observed in emulsions containing chia protein. In both scenarios, the particle size measurement indicates that the resultant emulsion does not qualify as a nanoemulsion. Thus, the efficacy of this protein in the context of obtaining nanoformulation, is unsatisfactory.

Another important parameter characterizing emulsions is the PDI. In our study, PDI results ranged from 0.280 (for nanoemulsions with whey protein) to 0.550 (for those with chia protein). The protein type significantly influenced the PDI value. Both for emulsions with sunflower and hemp oil, whey protein registered the lowest PDI values, specifically 0.280 and 0.317. For nanoemulsions anchored on plant proteins, those formulated with pea protein showcased superior stability (evidenced by a lower PDI value) compared to those with soy protein. Consistent with particle size trends, chia protein-emulsified oil registered the highest PDI value, being 0.550 for hemp oil and 0.442 for sunflower oil.

Incorporating protein as an emulsifier, aiming to reduce the interfacial tension at the oil–water boundary, led to smaller emulsion droplet sizes. However, an excessive protein concentration might cause a less stable system due to decreased interfacial elasticity of the droplets [17]. A low PDI value indicates a more homogeneous sample, which, concerning emulsions (particularly their stability), is sought after. The parameter’s value for all studied nanoemulsions was under 0.700. This is noteworthy as it implies a tight particle size distribution for the resulting nanoemulsions. Thus, there is not a large dispersion of droplet sizes within the sample [20].

### 2.2. Water Activity

Water activity is a key parameter that defines the microbiological stability of the products. Measurement results for vacuum drying (VD) and freeze drying (FD) nanoemulsions are presented in Table 2. Obtained value ranged from 0.059 to 0.244, indicating high microbiological stability of powders. Both the type of emulsifier used in nanoemulsion production, as well as the drying technique used, significantly influenced the value of the final water activity score. FD nanoemulsions had a slightly but statistically significant lower water activity score compared to their VD counterparts. Notably, FD emulsions made from animal proteins had statistically higher water activity measurements than those derived from plant proteins, which had values below 0.100.

Furthermore, the type of oil also played a role. FD nanoemulsions with sunflower oil paired with plant proteins exhibited lower water activity compared to hemp oil. In contrast, emulsions containing hemp oil and whey protein/whey protein isolate (0.181 and 0.165, respectively) had lower water activity compared to those that paired the same proteins with sunflower oil (0.227 and 0.192). Among VD nanoemulsions, formulations composed of pea protein had the lowest water activity, while whey protein isolate had the highest (0.107 and 0.244, respectively). Bajaj et al. [21] reported higher water activity in spray-dried nanoemulsions made of pea protein. Specifically, a value of 0.180 was consistent across all prepared emulsions, each of which incorporated three different types of pea protein preparations. In contrast, Smułek et al. [22] in fresh nanoemulsions containing hemp seed oil, whey protein, and Aesculus hippocastanum L. extract, water activity exceeded 0.75, with the value being contingent upon the proportions of the individual emulsion components. On the other hand, Teo et al. [23] developed nanoemulsions using spray-dried corn oil, whey protein isolate, and a coating material (trehalose, maltodextrin, or trehalose:maltodextrin blend), in which water activity values ranged from 0.19 to 0.39.

### 2.3. Color of the Emulsion

The results of the emulsion color measurement using the CIE L*a*b* system can be found in Table 2. The L* parameter values for FD emulsions ranged from 63.63 (for the chia protein emulsion) to 88.71 (for the emulsion with whey protein isolate). Meanwhile, the L* values for VD emulsions ranged from 66.76 (pea protein combined with hemp oil) to 82.46 (whey protein isolate paired with sunflower oil). Emulsions based on sunflower oil (both after FD and containing plant-based proteins) exhibited lower parameters compared to similar emulsions made with hemp oil. FD emulsions incorporating whey protein or whey protein isolate were the brightest. In contrast, those with chia protein were the darkest. A comparable trend was observed for VD emulsions: products with plant proteins were darker than those using animal proteins.

All VD emulsions exhibited a higher value of the a* parameter compared to the FD emulsions. For emulsions containing whey protein subjected to VD, the parameter in question was three times higher, and it was 1.5 times higher for formulations with pea protein. This reflects a more pronounced red color in the powders after VD. Nevertheless, in both scenarios, a reduced concentration of packed carotenoid compounds was observed when compared to the FD counterparts. This suggests that the intensified reddish proportion of powder color after VD may be attributed to the higher temperatures of VD. Such drying may contribute to stronger protein denaturation during heating and the formation of a surface moisture barrier. In addition, the drying method can influence the resulting powder’s color. Protein extracted from *Rana chensinensis* after FD displayed a distinctly yellow color, whereas when VD, it was brown. The product after FD had larger particle sizes, resulting in more pigment presence, giving the impression of a more yellow color. The extended that high temperatures used in VD might instigate Maillard reactions between amine residues and carbonyl compounds, leading to alterations in product coloration [24]. The lowest values of the discussed parameter were associated with emulsions made of animal protein while the highest values were found in emulsions with pea protein (averaging around 1.15 for freeze-dried emulsions and 4.00 for vacuum-dried emulsions). In contrast, statistically, the highest values were found in emulsions with pea protein, tallying 4.05 and 6.03 for FD and VD emulsions, respectively.

All dried emulsions showcased a positive value for the b* parameter, indicating a higher proportion of yellow color. Emulsions containing VD whey protein or whey protein isolate displayed the highest b* parameter values (41.25 and 41.05, respectively). As a consequence of VD, the b* factor value surged, with the most significant increase observed for emulsions with whey protein (around a 20% increase compared to FD). Formulations integrating hemp oil and whey protein exhibited higher values than those with sunflower oil.

Bajaj et al. [21] produced emulsions with three distinct pea proteins combined with flaxseed oil, which were then spray-dried. The resulting powders had L* (73.5–76.7), a* (2.8–4.9), and b* (18.6–27.7) parameter values. The L* and a* parameter outcomes are similar to our findings. However, the b* parameter, which indicates the yellow color proportion, was lower than our results. This elevated b* parameter in our study might stem from the addition of carotenoid compounds in the oil or from the distinct drying methods employed (i.e., freeze drying and vacuum drying). Conversely, Jarzębski et al. [17] noted in their research that using pea protein in an emulsion with hemp oil yielded fresher formulations that were lighter and more yellow. The results of the a* parameter obtained by the authors indicate a higher proportion of blue buffalo than red buffalo. On the other hand, the measurement of the b* factor was about three times lower (10.31) than in our study. Lastly, Smułek et al. [22] in their study on nanoemulsions containing hemp oil and whey protein isolate, observed that higher oil concentrations led to a decrease in the L* parameter value. Conversely, a larger whey-to-oil ratio seemed to push the color spectrum more toward the yellow side.

### 2.4. Identification and Quantification of Carotenoids and Tocopherols by UPLC-PDA LC-MS-Q/TOF

In this study, we identified 19 carotenoids (Table 3) in marigold flowers. The total carotenoid content in dried marigold flowers was 27 times lower than in the powder derived from isolated carotenoid compounds (Table 4), with values of 160.07 mg/100 g and 4327.81 mg/100 g, respectively. Depending on the flower’s cultivar and color, fresh petal carotenoid content can vary between 48.2 mg/100 g and 276.0 mg/100 g [25]. In the purified marigold compounds, the predominant carotenoid group was lycopene and its derivatives. Conversely, β-carotene emerged as the second most dominant compound in the mixture (accounting for approximately 15%) and the dominant compound among all carotenes. Analyzing the detailed compound profile in marigolds and their respective contents, the carotenoids were ranked as follows: (5Z,9Z,5′Z)-lycopene >> β-carotene >> (5Z,9Z,5′Z,9′Z)-lycopene >> lutein-5,6-epoxide >> lutein >> flavoxanthin >> violaxanthin. Our study’s findings, particularly concerning the dominant carotenoid compounds, mirror those reported for dried marigold flowers. In a study by Kishimoto et al. [26], fresh marigold flowers predominantly contained flavoxanthin. However, Pintea et al. [25] found varying dominant compounds across different fresh pot marigold flower petal varieties, with β-carotene or flavoxanthin appearing most often. The difference in the profile of carotenoid compounds might be attributed to the utilization of dried flowers (as opposed to fresh) and differences in plant varieties. Furthermore, the adoption of cutting-edge measurement techniques with enhanced detection capabilities contributed to the detection of low concentration compounds.

The prepared emulsions exhibited varying concentrations of carotenoid compounds (Table 4). The emulsion made up of pea protein isolate and hemp oil (after FD) showcased the highest carotenoid content. In contrast, the emulsion produced from whey protein and sunflower oil, when VD, displayed statistically the least content, registering 39.74 mg/100 g and 2.18 mg/100 g, respectively. Generally, VD emulsions exhibited significantly reduced carotenoid levels compared to their FD emulsions. Such a difference can be attributed to the higher temperatures encountered during VD, which might promote the degradation and isomerization (structural modification) of carotenoids [10]. Emulsions featuring pea protein showed a notably significantly higher carotenoid content compared to other formulations. This suggests that this specific emulsifier might possess the highest capacity to encapsulate bioactive ingredients, especially carotenoids. Observations also indicated that for formulations based on pea protein isolate (39.74 mg/100 g) and whey protein (9.26 mg/100 g), those incorporating hemp oil had a significantly higher content of carotenoid content than sunflower-based nanoemulsion (29.21 mg/100 g and 7.75 mg/100 g, respectively). Conversely, for nanoemulsions with chia protein and soy protein, mixtures utilizing sunflower oil (12.29 mg/100 g and 8.74 mg/100 g, respectively) displayed a significantly higher concentration of these bioactive compounds compared to hemp oil emulsion (12.29 mg/100 g and 8.74 mg/100 g, respectively). These patterns imply that various proteins might have distinct reactions when paired with particular oils, leading to potential interactions between the individual components of the emulsion.

In all emulsions, β-carotene was the dominant compound. Depending on the emulsion type, its amount varied from 15.72 mg/100 g (PP/HO/FD) to 0.42 mg/100 g (WP/SO/VD), translating to approximately 39.5% and approximately 19% of the total β-carotene content, respectively. In the purified carotenoid preparation, this compound made up about 15.5% of the total carotenoids and was the second dominant compound in marigolds. β-carotene is one of the most common carotenoids in foods, with the highest provitamin A activity. Similar to α-carotene and β-cryptoxanthin, β-carotene can be converted into retinol in humans [7]. It underscores the importance of supplementing the body with these compounds. It is also necessary to consider carotenoids, which do not exhibit properties typical of provitamin A, because this group is characterized by high antioxidant properties, affecting the neutralization of free radicals in the body, preventing the appearance of chronic diseases [27]. Even though lutein or its derivative was the primary compound in the carotenoid preparation, β-carotene dominated in the nanoemulsions. This suggests significant lutein degradation during emulsion preparation or its drying. Kuang et al. [28] revealed this compound’s instability, particularly its susceptibility to oxidative or thermal degradation. The chosen emulsifier can sway the final compound profile since the bioactive material’s behavior in the emulsion depends significantly on it. Emulsions using pea and chia proteins had more carotenoids than those with whey protein and its isolate. Rehman et al. [9] demonstrated that whey protein isolate enhances β-carotene’s oxidative stability, hinting that other proteins such as soy protein isolate, chia protein, and particularly pea protein isolate, might have comparable stabilizing properties.

8′R-luteoxanthin, violaxanthin, luteo-5,6-epoxide, flavoxanthin, lutein, and α-cryptoxanthin were undetectable in all prepared emulsions, despite their presence in both carotenoid preparations and marigold petals. The highest zeaxanthin concentrations were found in the PP/HO/VD (0.62 mg/100 g) and PP/HO/FD (0.25 mg/100 g) formulations. In the variants with pea protein isolate and sunflower oil, the compound was at 0.19 mg/100 g, regardless of the drying method. No zeaxanthin was detected in the whey protein and sunflower oil formulation. Antheraxanthin was absent in WPI/HO/FD and SP/HO/FD, but the highest concentration was in PP/HO/FD. The lutein derivative ((9Z)-lutein) only appeared in the pea protein isolate and hemp oil formulation after VD. This variant had a 0.22% concentration, about four times less than in the carotenoid preparation. (5ʹZ,9ʹZ)-rubixanthin was in trace amounts in two nanoemulsions (WPI/HO/VD and PP/HO/VD). (5Z,9Z,5′Z,9′Z)-lycopene and (5Z,9Z,5′Z)-lycopene were in all formulations, with the highest in the PP/HO/FD version (3.85 mg/100 g and 5.37 mg/100 g). In relation to the carotenoid preparation from marigold flowers, the (5Z,9Z,5′Z,9′Z)-lycopene was statistically significantly more (8.4%), while (5Z,9Z,5′Z)-lycopene was less (around 21%). Pea protein emulsions had higher concentrations of these compounds than other formulations, and VD materials had a higher percentage of these compounds compared to their FD counterparts. Trace δ-carotene amounts were detected in emulsions with whey protein isolate and pea protein isolate. In other instances, this carotene was not detected. Conversely, α-carotene was found solely in nanoemulsions made from pea protein. Depending on the oil and drying method used, its proportion in the specific variants ranged from 1.23% (PP/SO/FD) to 1.90% (PP/SO/VD) relative to the total compound content. γ-carotene appeared in small amounts across all emulsions, with its peak concentration found in the PP/HO/FD variant at 0.08 mg/100 g. This formulation also had the highest level of (5′Z)-γ-carotene, which was not detected in emulsions based on soy protein isolate. ϵ-carotene, the second most prevalent carotene in the preparation, had its maximum presence in the PP/HO/FD variant at 0.58 mg/100 g. This constituted a 1.46% contribution concerning the overall carotenoid content of that sample. For other versions containing pea protein isolate, ϵ-carotene had a comparable percentage relative to other compounds.

The results indicate that the ultimate concentration of carotenoid compounds in formulations is influenced by several factors, including the type of oil, drying method, and protein type. Drying methods have a significant impact on the profile and concentration of compounds, but the type of protein seems to be even more crucial. The choice of emulsifier also plays a role, as evidenced by the higher carotenoid content in formulations containing pea protein isolate. The selection of the appropriate oil can further influence the final compound profile. Emulsions based on hemp oil, for example, displayed increased levels of β-carotene, lycopene derivatives, and zeaxanthin. These hemp oil-based emulsions also had the highest carotenoid content overall.

Marigold flowers contain four types of tocopherols and four tocotrienols (δ, β, γ, and α) as presented in Table 4. As in the case of carotenoids, the obtained preparation showed a higher content of the tested compounds (about 27 times more) in relation to dried marigold petals. The obtained results are 56.29 mg/100 g and 2.07 mg/100 g, respectively. The main compound found in marigold flowers and its isolate was α-tocoferol (66% and 73% of all compounds, respectively). This substance, which belongs to the vitamin E family, is characterized by strong antioxidant properties [8]. Furthermore, α-tocopherol has various health benefits, including reducing the risk of vascular diseases and diabetes [14].

In the studied nanoemulsions, the sample containing pea protein and sunflower oil, after VD, had the highest content of tocotrienols and tocopherols (3.69 mg/100 g). The predominant tocopherol varied depending on the sample composition. α-Tocopherol dominated only in samples with whey protein or its isolate in combination with sunflower oil. Notably, only four emulsions (WPI/HO/FD; WP/SO/FD; WPI/HO/VD; and WP/SO/VD) had detectable amounts of tocotrienols, specifically α-tocotrienol. The tocopherol and tocotrienol content can be optimized with the choice of oil. Different oils and emulsifiers can determine the profile of encapsulated beneficial compounds. Occhiuto et al. [29] showed that hemp seed oil is a good source of γ-tocopherol (>70 mg/100 g). A similar relationship was observed in hemp oil-based emulsions, in which γ-tocopherol was the dominant tocopherol. Nanoemulsions that underwent lyophilization drying, which contained both whey protein and whey protein isolate combined with sunflower oil, exhibited a higher content of α-tocopherol than those with hemp oil. On the other hand, in the VD variants, the relationship described above was present only in the material made with whey protein and pea protein, but not with whey protein isolate.

There are reports that α-tocopherol protects β-carotene from radical autoxidation. Probably, the presence of α-tocopherol played a key role in preserving β-carotene in the formulated samples, protecting it from significant degradation. Conversely, when γ-tocopherol and β-carotene are combined, it can reverse the pro-oxidative effects of β-carotene, thereby enhancing the antioxidant potency of γ-tocopherol. The relationship between these compounds is intricate. This complexity also stems from β-carotene’s dual capabilities: it can both neutralize free oxygen radicals and mend tocopherol radicals. These tocopherol radicals emerge from the depletion of γ-tocopherol’s oxygen scavenging activity [30].

### 2.5. Biological Activity (Antioxidant and Anti-Diabetic Properties)

The antioxidant activity of plant-based products is significantly influenced by their bioactive compound content. In protein emulsions infused with marigold flower phytochemical extract, a notable correlation was observed between the presence of tocopherols, tocotrienols, and antioxidant capacity.

The results of antioxidant activity are presented in Table 5. The antioxidant activity measured by the ORAC method ranged from 0.20 mmol Trolox/100 g (PP/HO/FD and WP/HO/FD) to 0.65 mmol Trolox/100 g (WPI/HO/VD). Hemp oil-based emulsions after VD exhibited superior antioxidant activity compared to their FD emulsions. It is plausible that the elevated temperatures used during VD facilitated interactions between proteins (or their components) and the bioactive compounds in the hemp oil. This might lead to the formation of a complex with enhanced antioxidant properties. In contrast, for emulsions made with sunflower oil, no statistically significant difference (*p* ≤ 0.05) was observed in terms of the drying method. The sole exception was the formulation incorporating whey protein isolate, which displayed increased activity. Given its lower oxidative stability, sunflower oil is more prone to oxidation. Encapsulating the oil, which contains carotenoid compounds, within a water–protein structure effectively shielded the material from the adverse impacts of high drying temperatures. This underscores the importance of obtaining nanoemulsions to preserve the antioxidant properties of the material dissolved in oil. Despite this, the oil can be also a source of antioxidants. Occhiuto et al. [29] demonstrated in their study that hemp oil has a high oxygen radical absorption capacity. Marigold also stands out as a rich source of antioxidants. Villalva et al. [31] compared the antioxidant activity (ORAC) across three techniques of extracting bioactive ingredients from pot marigold flowers. They observed values spanning from 38 to 132 mmol Trolox/100 g. This variance in antioxidant values might stem from the presence of distinct bioactive compounds in the extract—primarily polyphenolic compounds, as opposed to carotenoid compounds in our experiment.

The results derived from the ABTS cation radical scavenging capacity deviate from those obtained via the ORAC method. The emulsion formulated from whey protein isolate and hemp oil (after VD) showcased the highest antioxidant activity, registering at 0.63 mmol Trolox/100 g. Conversely, the lowest value was observed in the emulsion comprising whey protein and sunflower oil after VD (0.13 mmol Trolox/100 g). For the remaining emulsions, statistical differences were nonexistent (*p* ≤ 0.05).

Liu et al. [32] revealed a lack of significant variance in the ABTS antioxidant activity results across nanoemulsions made of whey protein isolate, soy protein isolate, brown rice protein isolate, or peanut protein isolate. This observation formulated by the cited authors are consistent with the results presented in this publication, suggesting that the protein type incorporated does not heavily influence antioxidant activity when accessed via the ABTS method. The observed uptick in antioxidant activity might be attributed to the Maillard reaction, which manifests under elevated temperature conditions, as elucidated by Liu et al. [33].

The observed discrepancies in values obtained from the ABTS and ORAC methods can largely be attributed to their distinct reaction mechanisms. The ORAC method operates on the principle of hydrogen atom transfer, offering a broader potential for determining antioxidant capacity across a larger group of compounds. In contrast, the ABTS method focuses on the transfer of a single antioxidant electron to the oxidized compound, which lends specificity to this method for particular compounds. Despite these differences, a consistent correlation was observed between the antioxidant activity and the presence of tocopherols in both tests.

The enzymes α-amylase and α-glucosidase are primarily responsible for breaking down starch into oligosaccharides. From these, glucose molecules are released and then absorbed into the bloodstream. Consequently, inhibiting these enzymes can play a significant role in diabetes prevention [34]. In our studies, all the tested emulsions demonstrated a stronger inhibitory effect on α-amylase than on α-glucosidase, as shown by the lower values presented in Table 5. The α-amylase inhibitory range spanned from 487.70 mg/mL to 1672.28 mg/mL for FD emulsions with sunflower oil containing whey protein isolate and those with chia protein, respectively. We observed that VD emulsions using sunflower oil exhibited approximately 30% higher α-amylase inhibition capabilities compared to those containing hemp oil. When it came to lyophilized nanoemulsions, all formulations made of protein isolate and sunflower oil (including whey protein isolate, soy protein isolate, and pea protein isolate) showed twice the inhibitory potency against α-amylase compared to those made with hemp oil.

A study by Wu et al. [34] using spray-dried and lyophilized powders made of whey protein isolate and an anthocyanin-rich concentrate from blackcurrant proposed that FD could be more effective in preserving α-amylase inhibitory activity compared to VD. This outcome was likely due to the milder temperatures in FD, preserving a higher quantity of bioactive compounds that have antidiabetic properties. Interestingly, we did not observe this phenomenon in our study. The carotenoids we extracted from marigolds for oil fortification are more thermally stable than anthocyanins. This suggests that when creating formulations based on carotenoids, the temperature might not be as critical a factor as it is for compounds such as anthocyanins. In addition, proteins can exert an inhibitory effect on digestive enzymes. This property allows them to moderate starch digestion by inhibiting the activity of the α-amylase enzyme. Zhang et al. [35] reported such inhibitory activity for whey protein isolate. Similarly, Chen et al. [36] demonstrated that both soy protein isolate and wheat gluten protein can delay the digestion of starchy foods. Among the two, gluten protein exhibited a more pronounced inhibitory capacity against α-amylase. However, incorporating various factors—such as differing drying methods, types of oils, and protein sources—led to statistically varied results. This variance suggests potential interactions between the individual components of the nanoemulsion during the drying process, which could subsequently influence the formation of either stronger (as in WPI/SO/FD) or weaker (as in CHP/HO/FD) inhibitory properties of the final material.

On the other hand, the most potent α-glucosidase inhibitory activity, expressed as IC50, was observed for the VD emulsion with pea protein isolate and hemp oil, and the FD emulsion with chia protein and hemp oil, with values of 1630.44 and 1640.65 mg/mL, respectively. Conversely, the least inhibitory activity was found in the formulation using whey protein isolate and sunflower oil (post sublimation drying) with a value of 2729.48 mg/mL. The FD emulsions generally demonstrated a lower α-glucosidase inhibitory capacity (indicated by higher IC50 values) compared to those subjected to VD. An exception was the formulation made of whey protein isolate and hemp oil, which had IC50 values of 2338.89 and 2503.79 mg/mL for sublimation and VD methods, respectively. Notably, the majority of emulsions formulated with sunflower oil had significantly weaker inhibitory effects against α-glucosidase than those with hemp oil. Moreover, a correlation was observed between enhanced α-glucosidase inhibition and a higher carotenoid compound concentration in the emulsions, a finding supported by PCA analysis. For instance, Qi and Kim [37] demonstrated that (all-E)-lutein and (9-Z)-zeaxanthin, isolated from the C. ellipsoidea green alga, inhibited α-glucosidase sourced from S. cerevisiae. In contrast, Nimbalkar et al. [38] found that β-carotene lacks significant inhibitory properties concerning this enzyme. The varied results suggest that the choice of oil and drying technique influences outcomes more than protein type.

### 2.6. Principal Component Analysis (PCA)

**Figure 1:**
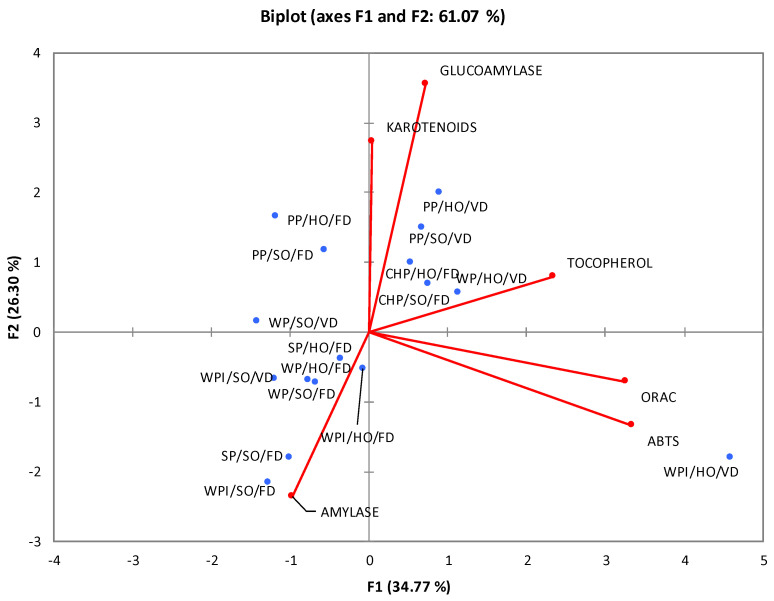
Principal component analysis for the tested nanoemulsions with *Calendula officinalis* L. phytochemical extract.

Performing PCA (Figure 1) helped us elucidate the relationships between various properties of the studied emulsions. A strong correlation between variables responsible for antioxidant activity (ABTS and ORAC) is evident, as indicated by the small angle between their vectors. This relationship boasts a high correlation coefficient (r = 0.789). However, there was no positive correlation between the antioxidant properties and the high concentration of carotenoid compounds. This observation might stem from the minimal presence of substances not categorized under compounds with provitamin A properties, which are renowned for their robust antioxidant activity [27]. On the other hand, a positive correlation was observed between the presence of tocopherols and antioxidant abilities. Emulsions richer in tocopherols exhibited enhanced antioxidant potential. This correlation aligns with the widely recognized strong antioxidant properties of tocopherol, especially α-tocopherol, which is a component of vitamin E. The results hint at the influence of specific compounds on antioxidant properties, rather than the collective impact of an entire group of compounds. Another discernible correlation was between the content of carotenoid compounds and the ability to inhibit α-glucosidase, as represented by the second component. VD pea protein emulsions (PP/HO/VD and PP/SO/VD) and chia protein emulsions (CHP/SO/FD and CHP/HO/FD) generally exhibited higher carotenoid content and superior α-glucosidase inhibition compared to the average.

The negative correlation was observed between α-amylase and α-glucosidase inhibition abilities in the tested nanoemulsions, with a correlation coefficient of r = −0.395. Evaluating the position of individual emulsions on the graph reveals that samples with heightened α-amylase inhibition capabilities were predominantly FD. However, the majority of VD emulsions, such as the WPI/SO/FD emulsion, showcased better α-glucosidase inhibition. A notable exception was the WPI/HO/VD emulsion, which, despite its significantly lower-than-average carotenoid content and marginal α-glucosidase inhibition, displayed potent α-amylase inhibition. The PCA analysis indicated that there is no strong relationship between the type of oil used and the individual functional properties. Yet, graphically, there appeared to be a cluster of points representing specific emulsions, characterized by identical emulsifiers (proteins) and properties. This suggests the type of protein may influence the health profile of the material. Notably, variants based on pea protein isolate exhibited the most advantageous functional properties, signifying the highest efficiency of this material in forming carotenoid nanoemulsions.

The obtained research allowed us to determine the emulsification process conditions using plant and animal proteins. The obtained results will help to assess the appropriateness of individual emulsifiers concerning the optimal physical parameters of emulsions and their health-promoting benefits. In addition, the development of the final formulation will allow indication of the pro-health potential of the obtained complex and reveal possible interactions among its individual components. Additionally, it will allow for selection of the best emulsifying material in terms of the best functional properties.

## 3. Materials and Methods

### 3.1. Reagents

Sunflower and hemp oil were purchased from a supermarket located in Poland. Whey protein isolate, whey protein, pea protein isolate, and soy protein were purchased from MyProtein (Manchester, UK). Chia protein was purchased from Purasana (Wevelgem, Belgium). Standards for carotenoid compounds were purchased from Extrasynthese (Genay, France). All reagents essential to determination of biological activity, and those required for LC-MS purity ultraperformance liquid chromatography were purchased from Merck (Darmstadt, Germany). Reagent to extraction procedure: NaCl, ethanol solution 96% cz.d.a. and ethyl acetate cz.d.a. were purchased from P.P.H “StanLab” sp. z o.o. (Lublin, Poland), potassium hydroxide from “Chempur” (CAS 1310-58-3, Piekary Śląski, Poland), and n-hexane czda-basic 99% from “Chempur“(Piekary Śląskie, Poland).

### 3.2. Plant Material

The primary ingredient utilized was isolated carotenoid compounds from pot marigold flowers. To prepare this, dried marigold flowers, procured from a local store, were immersed in water until an extract with a concentration of 0°Bx was achieved. The plant material was then frozen, subjected to lyophilization, and subsequently ground into a fine consistency. This prepared raw material was employed in the extraction process, which is delineated below for obtaining phytochemical extract from marigold

In summary, a premeasured pot marigold sample (6 g) was poured in ethanol (110 mL), fortified with 0.05% BHT, and combined with a 60% KOH solution (12 mL). This mixture was then situated in a water bath (80 °C) and agitated continuously for an hour. Post this duration, a 1% NaCl solution (15 mL) was introduced to the mix, along with an extraction mixture (10 mL) comprising hexane and ethyl acetate (9:1 ratio). The resultant mixture was shaken for another hour with 260 rpm (ELMI, Orbital shaker DOS-10L, Riga, Latvia), followed by centrifugation (15 °C, 4500 rpm, 5 min). The ensuing upper layer was transferred to a separate vessel, and the residue was poured again with the extraction mixture. This entire procedure was reiterated four times. The extracts gathered from all these iterations were evaporated (Hei-VAP Expert Control; Heidolph, Schwabach, German), and the remaining substance was lyophilized by a FreeZone freeze dryer (Labconco Corp., Kansas, MO, USA), for 20 h (collector temperature of −60 °C, shelf temperature of +24 °C, vacuum 0.080 mbar)

### 3.3. Procedure of Preparing the Nanostructures

The procedure for preparing nanostructures included the following three steps: (i) Dissolving the isolated carotenoid compounds (phytochemical extract from marigold) in oil. (ii) Producing nanoemulsions through sonication. (iii) Drying the obtained nanostructures. Initially, sunflower and hemp oils were prepared, containing 3% (*w*/*v*) phytochemical extract from marigold. To achieve this, 1.5 g of extract was added to 50 mL of oil and mixed using a magnetic stirrer (16 h, 800 rpm). Subsequently, an oil-in-water (o/w) emulsion was formulated. For this purpose, either plant or animal protein was mixed with water using a magnetic stirrer (1 h, 800 rpm). The appropriate oil was then added in a 90:10 mass ratio. The mixed solution was homogenized for 4 min at 5.0 × 1000 rpm, followed by sonication with an Ultrasonic processor VCX 500 (from Sonics & Materials, Newtown, CT, USA). The operating conditions were set at an amplitude of 50% and an operating mode of 30 s on, followed by a 30 s pause. For a sample with a total volume of 25 mL, the sonication process lasted 15 min, and the system was constantly cooled. Depending on the raw material, the protein concentration in water ranged from 7.5% (for chia protein, pea protein isolate, and soy protein isolate) to 10% (for whey protein and whey protein isolate). The concentrations for individual proteins were determined based on optimization for each emulsifier, as established during laboratory tests.

In this way, 10 liquid emulsions based on 5 proteins and 2 types of oil were obtained, which were then dried using two methods: vacuum drying (VD) and freeze-drying (FD). The exceptions were formulations based on chia protein and soy protein, which were subjected to freeze-drying only.

VD was performed using a Vacucell 111 Eco Line (MMM Medcenter Einrichtungen GmbH, Planegg, Germany) for 20 h at 60 °C with pressure 0.1 mbar. FD, using the FreeZone freeze dryer (Labconco Corp., Kansas, MO, USA), was also conducted for 20 h with the parameters set at a collector temperature of −60 °C and a shelf temperature of +24 °C. The resultant powders were then crushed in a mortar and vacuum sealed. In this way, 16 variants of dried emulsions were finally obtained.

### 3.4. Particle Size and Polydispersity Index (PDI)

The emulsion particle size and polydispersity index (PDI) were measured using a SZ-100-Z2 nanoPartica instrument (Horiba, Kyoto, Japan) based on the dynamic light scattering (DLS) method in temperature +25 °C. Each sample was diluted ten times in distilled water before measurement (0.4 mL of sample to 3.6 mL of water). The measurement was performed in triplicate (n = 3) and expressed as the average of measurements.

### 3.5. Water Activity (a_w_)

Water activity measurements of the dried nanostructures were taken using a device from LabParnetr (Novosina, Lachen, Switzerland). Approximately 0.500 g of the sample was placed into the measuring vessel. The sample was measured thrice (n = 3), and the results are presented as the average of these measurements (±standard deviation).

### 3.6. Color by CIE L*a*b*

The color of the dried emulsions was analyzed using a CR-400 spectrophotometer (Konica Minolta Inc., Tokyo, Japan). The results are presented in CIE L*a*b* space and expressed as the mean of the measurements (n = 3) ± standard deviation.

### 3.7. Extraction Procedure, Identification, and Quantification of Carotenoids Compounds by LC-MS-Q/TOF and UPLC-PDA

Approximately 3 g of the powdered nanoemulsion was combined with 7.5 mL of ethanol containing 0.05% BHT and mixed intensively. Subsequently, 1.5 mL of a 60% KOH solution was added. The mixture was then agitated in a Hydro H20 SOW water bath (Lauda, Germany) at 80 °C for an hour. After the saponification process and once the sample cooled, both 10 mL of a 1% NaCl solution and 10 mL of an extraction mixture made up of hexane: ethylacetate (9:1 ratio) were introduced. This mixture was then placed in a rotator (Rotator Multi RS-60, Biosan, Riga, Latvia). After 40 min of rotation, the top layer of the solution was separated. This extraction step with the reagent was performed three times in total. The upper layers obtained from all the extractions were combined and evaporated to dryness using the XcelVap^®^ (Thermo Scientific, Salem, MA, USA) under a nitrogen atmosphere. The residue was redissolved in 1.5 mL of methanol containing tetrahydrofuran. The prepared sample was set aside for subsequent analysis.

Quantitative analysis of carotenoids was executed as per the method detailed by Turkiewicz et al. [39]. For the identification and quantification of carotenoids, 10 μL of each sample was examined using a BEH RP C18 column (2.1 × 100 mm, 1.7 μm; Waters Corp., Drinagh, Ireland) maintained at 32 °C, employing gradient elution at a flow rate of 0.5 mL/min over a duration of 16.60 min. The eluents used were solvent A (0.1% formic acid) and solvent B (acetonitrile:methanol in a 7:3 ratio, *v*/*v*). Characterization of the individual components was accomplished through MS/MS analysis, retention time, spectral data, and references from prior literature (Pintea et al. [25]; Kishimoto [26]; and Balázs et al. [40]). The carotenoids compounds were determined based on available chromatograms of identified carotenoid, comparing retention times (min) as well as molecular ions (MS/MS) and fragment (*m*/*z*) values, and maximum wavelength of absorption (detection wavelengths for carotenes were 448–492 nm and for xanthophhyls were 416–480 nm), and using reference standards such as: zeaxanthin, violaxanthin α- and β-cryptoxanthin, lutein, lycopene, and also α-, β-, γ-, and δ-carotene.

Results are presented in terms of mg per 100 g of the product.

### 3.8. Identification and Quantification of Tocotrienols and Tocopherols by LC-MS-Q/TOF and UPLC-PDA

The sample for analysis was prepared following the procedure outlined above. Qualitative and quantitative assessments of tocopherols and tocotrienols were executed based on the method delineated by Turkiewicz et al. [39]. In summary, 5 μL of the sample underwent analysis on an ACQUITY UPLC BEH Shield RP C18 column (1.7 µm, 2.1 × 100 mm; Waters Corp., Milford, CT, USA) maintained at 30 °C. Isocratic elution was employed at a flow rate of 0.45 mL/min over a duration of 12 min. The eluents used comprised solvent A (methanol) and solvent B (0.1% *v*/*v* aqueous solution of formic acid) in an 88:12 (*v*/*v*) ratio. The excitation wavelength was set at 290 nm, while the emission wavelength was at 330 nm. Individual component identification and characterization were accomplished using reference standards and calibration curves created from α-tocopherol, β-tocopherol, γ-tocopherol, δ-tocopherol, and α/β/γ/δ-tocotrienols. Results are presented in terms of mg per 100 g of the product.

### 3.9. Biological Properties: Antioxidant Activity (ABTS, ORAC) and Ability to Inhibit α-Amylase and α-Glucosidase

The sample used for bioactivity evaluations was prepared in accordance with the carotenoid extraction procedure outlined earlier (see Section 3.7) with one modification—BHT was removed from all reagents to not affect the activity measurement.

The antioxidant activity was determined using two methodologies: the ABTS cation radical scavenging capacity and the oxygen radical absorbance capacity (ORAC). These methods were detailed by Re et al. [41] and Ou et al. [42], respectively. The ABTS measurement involved measuring the reduction in color intensity, which was inversely proportional to the antioxidant power. The analysis was performed by measuring the absorption of the sample mixed with the ABTS reagent after 6 min of reaction time at a wavelength of 734 nm. The ORAC assay was performed by measuring the decrease in fluorescence due to the presence of free radicals. For this purpose, phosphate buffer, 2,2′-azobis(2-amidinopropane) dihydrochloride, and fluorescein were added to the tested sample, then everything was mixed and measured every 5 min at wavelengths 493 nm and 515 nm (excitation and emission wavelengths, respectively). The positive control was Trolox and the negative control was methanol. The water was used instead of the sample for blank. The determined antioxidant activities were reported in terms of mmol Trolox per 100 g.

The antidiabetic activity, represented by the capacity to inhibit α-amylase and α-glucosidase in the powdered nanoemulsion, was assessed following the procedure set by Nowicka et al. [43]. Briefly, the inhibitory capacity of α-amylase was measured by changes occurring during the reaction of starch remaining after enzymatic hydrolysis with iodine in potassium iodide at a wavelength of 600 nm. In turn, α-glucosidase activity inhibitors were measured at a wavelength of 405 nm, and the measurement was based on the reaction of the enzyme with the β-d-glucosidase substrate. The positive control was acarbose and the negative control was methanol (the background was samples with buffer instead of the enzymes). Results are presented as IC50 values. During all measurements, temperature was 21 °C ± 1 °C.

### 3.10. Statistical Analysis

The results obtained, represented as mean values from three replicates (n = 3), were analyzed using the analysis of variance (ANOVA) and further subjected to Tukey’s test with the help of STATISTICA 13 (StatSoft, Krakow, Poland). Principal component analysis (PCA) was carried out utilizing XLSTAT (Addinsoft, New York, NY, USA).

## 4. Conclusions

The use of the emulsification process can be an interesting way of delivering bioactive compounds. Our research indicates that not all proteins (chia protein) are suitable for nanoemulsion production because of the high particle size results (<500 nm). Pea protein and whey protein proved to be the most suitable candidates for formulating nanoemulsions. The selection of the appropriate protein may shape the specific features and properties of the emulsion. The highest content of carotenoid compounds was determined in the pea protein-based nanoemulsion (39.74 mg/100 g). Our investigation revealed that nanoemulsions fortified with pea protein exhibited rich carotenoid compounds. This presence, intriguingly, did not correlate with potent antioxidant activity. Conversely, emulsions composed of whey protein manifested more robust antidiabetic activity, evidenced by their superior α-amylase inhibitory capacity compared to other samples. The highest inhibitory power had WPI/SO/FD emulsion (487.70 mg/mL). Additionally, health-promoting features can be shaped by selecting the appropriate drying technique and selecting the appropriate type of oil.

The research conducted revealed a minimal presence of lycopene and its derivatives in the encapsulated marigold carotenoid preparation. This underscores the need for additional research focused on safeguarding this compound against degradation. On the other hand, we observed high levels of β-carotene. This finding suggests that the employed method holds potential as an avenue for preserving the valuable properties of this compound.

## Figures and Tables

**Table 1:** Particle size distribution of nanoemulsion.

Lp.	Nanoemulsion Composition		Particle Size [nm]	Polydispersity Index (PDI)
	Type of Protein	Type of Oil		
1	Whey protein	Hemp Oil	194.23 ± 1.19 ^a^	0.280 ± 0.01 ^a^
2	Whey protein isolate		231.33 ± 28.83 ^bc^	0.380 ± 0.02 ^f^
3	Soy protein isolate		257.53 ± 8.50 ^c^	0.354 ± 0.04 ^d^
4	Pea protein isolate		265.13 ± 17.22 ^cd^	0.339 ± 0.04 ^c^
5	Chia protein		4598.80 ± 358.79 ^e^	0.550 ± 0.08 ^i^
6	Whey protein	Sunflower Oil	198.67 ± 12.08 ^ab^	0.317 ± 0.02 ^b^
7	Whey protein isolate		196.77 ± 3.13 ^a^	0.347 ± 0.03 ^d^
8	Soy protein isolate		254.50 ± 19.92 ^c^	0.386 ± 0.02 ^g^
9	Pea protein isolate		302.27 ± 22.15 ^d^	0.363 ± 0.02 ^e^
10	Chia protein		6799.10 ± 410.22 ^f^	0.442 ± 0.10 ^h^

The data shown are mean values ± SD (n = 3); different letters in the column mean statistically significant differences, based on Tukey’s test at *p* < 0.05.

**Table 2:** Water activity and color parameters of emulsions.

Sample			Water Activity		Colour Parameters	
Type of Protein	Type of Oil	Drying Technique		L*	a*	b*
Whey protein	Hemp Oil	Freeze drying (FD)	0.181 ^h^	82.38 ± 0.43 ^i^	1.38 ± 0.11 ^c^	31.95 ± 0.09 ^f^
Whey protein isolate			0.165 ^b^	84.73 ± 0.09 ^j^	0.72 ± 0.01 ^a^	30.02 ± 0.38 ^e^
Soy protein isolate			0.081 ^d^	80.70 ± 0.13 ^g^	1.76 ± 0.04 ^d^	25.00 ± 0.10 ^b^
Pea protein isolate			0.075 ^b^	74.62 ± 0.22 ^d^	3.90 ± 0.03 ^h^	33.75 ± 0.15 ^g^
Chia protein			0.100 ^f^	63.63 ± 0.10 ^a^	3.19 ± 0.05 ^e^	30.70 ± 0.15 ^e^
Whey protein	Sunflower Oil		0.227 ^k^	86.50 ± 0.27 ^k^	1.38 ± 0.14 ^c^	26.65 ± 0.12 ^c^
Whey protein isolate			0.192 ^i^	88.71 ± 0.04 ^l^	1.14 ± 0.03 ^b^	24.64 ± 0.07 ^a^
Soy protein isolate			0.079 ^c^	78.26 ± 0.08 ^f^	1.80 ± 0.01 ^d^	30.15 ± 0.15 ^e^
Pea protein isolate			0.059 ^a^	78.16 ± 0.13 ^f^	4.19 ± 0.05 ^i^	29.96 ± 0.23 ^e^
Chia protein			0.095 ^e^	71.29 ± 0.14 ^c^	3.29 ± 0.05 ^f^	28.69 ± 0.05 ^d^
Whey protein	Hemp Oil	Vacuum drying (VD)	0.185 ^hi^	74.28 ± 0,03 ^d^	4.28 ± 0.03 ^j^	41.25 ± 0.23 ^j^
Whey protein isolate			0.185 ^hi^	77.62 ± 0.07 ^e^	3.59 ± 0.03 ^g^	41.05 ± 0.17 j
Pea protein isolate			0.107 ^f^	66.76 ± 0.42 ^b^	5.65 ± 0.08 ^k^	36.07 ± 0.20 ^i^
Whey protein	Sunflower Oil		0.217 ^j^	81.45 ± 0.30 ^h^	4.43 ± 0.14 ^j^	34.58 ± 0.21 ^h^
Whey protein isolate			0.244 ^l^	82.46 ± 0.28 ^i^	3.73 ± 0.16 ^gh^	35.24 ± 0.40 ^h^
Pea protein isolate			0.114 ^g^	71.63 ± 0.51 ^c^	6.45 ± 0.30 ^l^	33.67 ± 0.32 ^g^

The data shown are mean values ± SD (n = 3). Different letters in the column mean statistically significant differences, based on Tukey’s test at *p* < 0.05.

**Table 3:** Carotenoids profile determinate in pot marigold (UPLC-PDA LC-MS-Q/TOF).

Number of Peak	Retention Time	Carotenoid	λ Min/Max (nm)	[M + H]^+^	MS/MS
1	5.67	(8′R)-luteoxanthin	399/422/447	601.5	583.5, 565.5, 509.5, 491.5, 221.1
2	5.78	violoxanthin	433/465	601.4	509.4, 491.4, 567.4
3	5.85	lutein-5,6-epoxide	415/436/465	585.4	567.1, 492.3, 244.9
4	5.91	flavoxanthin	391/443/468	585.4	567.1, 492,3, 244.9
5	6.18	lutein	447/473	569.4	551.4, 533.4;
6	6.36	zeaxanthin	447/476	568.9	550.9, 532.9, 476.4, 429.4
7	6.49	antheraxanthin	415/443/471	585	567, 549, 493, 221
8	6.59	(9Z)-lutein	441/469	536.9	550.9, 532.9, 476.4, 429.4
9	6.70	(5′Z,9′Z)-rubixanthin	448/477	552	551.4, 533.4;
10	6.80	α-cryptoxanthin	449/476	553	535, 495,461
11	8.00	(5Z,9Z,5′Z,9′Z)-lycopene	437/461/494	537	467, 444
12	8.07	(5Z,9Z,5′Z)-lycopene	442/463/490	537	467, 444
13	9.02	δ-carotene	429/467/497	537	481, 444
14	9.29	α-carotene	427/452/489	537	481, 444
15	9.45	γ-carotene	463/489	537	467, 444
16	9.55	(5′Z)-γ-carotene	429/459/486	537	467, 444
17	9.81	ϵ-carotene	417/443/470	537	467, 444
18	9.97	β-carotene	453/480	536.9	444.2; 430.3; 399.3
19	10.04	(*9-cis)-*β-carotene	449/477	536.9	444.2; 430.3; 399.3

**Table 4:** Content of carotenoid compounds, tocopherols and tocotrienols in marigold and its emulsions [mg/100 g].

Compounds	WP/HO/ FD	WPI/HO/ FD	SP/HO/ FD	PP/HO/ FD	CHP/HO/ FD	WP/SO/ FD	WPI/SO/ FD	SP/SO/ FD	PP/SO/ FD	CHP/SO/ FD	WP/HO/ VD	WPI/HO/ VD	PP/HO/ VD	WP/SO/ VD	WPI/SO/ VD	PP/SO/ VD	F	I
Carotenoids																		
(8′R)-luteoxanthin	nd	nd	nd	nd	nd	nd	nd	nd	nd	nd	nd	nd	nd	nd	nd	nd	0.05	1.27
violoxanthin	nd	nd	nd	nd	nd	nd	nd	nd	nd	nd	nd	nd	nd	nd	nd	nd	4.82	123.86
luteo-5,6-epoxide	nd	nd	nd	nd	nd	nd	nd	nd	nd	nd	nd	nd	nd	nd	nd	nd	3.94	197.87
flavoxanthin	nd	nd	nd	nd	nd	nd	nd	nd	nd	nd	nd	nd	nd	nd	nd	nd	4.82	123.86
lutein	nd	nd	nd	nd	nd	nd	nd	nd	nd	nd	nd	nd	nd	nd	nd	nd	2.09	191.46
zeaxanthin	0.13	0.07	0.03	0.25	0.09	nd	0.05	0.07	0.19	0.14	0.07	0.14	0.62	nd	0.07	0.19	0.86	10.14
antheraxanthin	0.04	nd	nd	0.30	0.04	0.07	0.06	0.07	0.17	0.06	0.07	0.03	0.08	0.03	0.09	0.03	0.65	28.59
(9Z)-lutein	nd	nd	nd	nd	nd	nd	nd	nd	nd	nd	nd	nd	0.07	nd	nd	nd	0.88	35.42
(5′Z,9′Z)rubixanthin	nd	nd	nd	nd	nd	nd	nd	nd	nd	nd	nd	0.03	0.04	nd	nd	nd	0.66	32.90
α-cryptoxanthin	nd	nd	nd	nd	nd	nd	nd	nd	nd	nd	nd	nd	nd	nd	nd	nd	0.03	0.82
(5Z,9Z,5′Z,9′Z)-lycopene	0.59	1.03	0.55	3.85	0.99	0.62	0.73	0.89	2.67	1.08	0.89	1.60	3.51	0.29	1.93	2.20	10.14	365.02
(5Z,9Z,5′Z)lycopene	0.81	1.65	0.68	5.37	1.03	0.82	1.22	1.20	3.62	1.43	1.20	2.21	5.06	0.47	2.87	3.89	65.18	920.41
δ-carotene	0.01	nd	nd	0.02	nd	nd	0.01	nd	0.02	0.01	nd	0.01	0.01	nd	0.01	0.01	0.07	3.27
α-carotene	nd	nd	nd	0.50	nd	nd	nd	nd	0.36	nd	nd	nd	0.44	nd	nd	0.46	0.70	31.16
γ-carotene	0.01	0.03	0.01	0.08	0.01	0.01	0.01	0.01	0.04	0.02	0.01	0.02	0.04	0.01	0.03	0.06	0.12	5.01
(5′Z)-γ-carotene	0.04	0.12	nd	0.23	0.07	0.06	0.04	nd	0.19	nd	nd	0.09	0.18	nd	0.10	0.11	0.44	15.71
ϵ-carotene	0.06	0.20	0.08	0.58	0.11	0.11	0.09	0.10	0.36	0.14	0.10	0.15	0.45	0.14	0.20	0.38	0.61	34.69
β-carotene	4.01	6.27	1.64	15.72	2.34	2.79	1.81	3.33	10.47	5.51	3.10	4.22	9.28	0.42	3.95	8.42	12.73	669.28
Others	3.26	4.60	3.84	12.84	3.27	3.26	3.31	3.07	11.11	3.92	2.82	3.91	11.24	0.83	7.25	7.97	51.28	1537.05
**Total**	**9.26 j**	**13.98 h**	**6.82 o**	**39.74 c**	**7.95 lm**	**7.75 m**	**7.33 n**	**8.74 k**	**29.21 e**	**12.29 i**	**7.58 mn**	**12.42 i**	**31.63 d**	**2.18 p**	**16.50 g**	**23.72 f**	**160.07 b**	**4327.81 a**
Tocotrienols and Tocopherols																		
δ-tocotrienol	nd	nd	nd	nd	nd	nd	nd	nd	nd	nd	nd	nd	nd	nd	nd	nd	0.05	1.72
β-tocotrienol	nd	nd	nd	nd	nd	nd	nd	nd	nd	nd	nd	nd	nd	nd	nd	nd	0.02	0.88
γ-tocotrienol	nd	nd	nd	nd	nd	nd	nd	nd	nd	nd	nd	nd	nd	nd	nd	nd	0.10	2.41
α-tocotrienol	nd	0.08	nd	nd	nd	0.02	nd	nd	nd	nd	nd	0.44	nd	0.04	nd	nd	0.04	1.81
δ-tocopherol	0.04	0.04	0.02	0.03	0.02	nd	nd	0.02	0.03	0.04	0.03	nd	0.06	nd	nd	0.05	0.04	0.44
β-tocopherol	nd	0.05	nd	0.08	0.03	0.08	0.05	nd	nd	nd	nd	0.09	0.12	0.03	0.04	nd	0.10	2.93
γ-tocopherol	1.68	1.46	1.17	0.36	0.82	0.05	0.06	0.83	0.69	1.79	0.96	1.49	2.53	0.04	0.10	3.32	0.34	4.88
α-tocopherol	0.23	0.55	0.29	0.31	0.16	1.40	0.90	0.11	0.22	0.06	0.29	0.79	0.12	0.64	0.60	0.32	1.38	41.22
**Total**	**1.95 f**	**2.18 e**	**1.48 i**	**0.78 o**	**1.08 k**	**1.55 h**	**1.01 l**	**0.96 m**	**0.93 n**	**1.89 g**	**1.28 j**	**2.81 d**	**2.83 d**	**0.75 p**	**0.74 p**	**3.69 b**	**2.07 c**	**56.29 a**

WP—whey protein; WPI—whey protein isolate; SP—soy protein isolate; PP—pea protein isolate; CHP—chia protein; HO—hemp oil; SO—sunflower oil; FD—freeze drying; VD—vacuum drying; F—flowers from pot marigold; I—isolated compounds from pot marigold; and nd—non detected. The data shown mean values (n = 3); different letters show statistical significance differences (ANOVA, Tukey’s post hoctest, *p* < 0.05).

**Table 5:** Antioxidant and anti-diabetic activity.

			Antioxidant Activity (mmol Trolox/100 g)		Anti-Diabetic Activity (IC_50_ [mg/mL])	
Type of Protein	Type of Oil	Drying Technique	ABTS	ORAC	α-Amylase	α-Glucosidase
Whey protein	Hemp Oil (HO)	Freeze drying (FD)	0.20 ± 0.08 ^bcd^	0.20 ± 0.01 ^h^	1042.77 ± 10.43 ^f^	2485.12 ± 37.28 ^h^
Whey protein isolate			0.26 ± 0.05 ^b^	0.24 ± 0.01 ^ef^	874.82 ± 8.75 ^de^	2338.89 ± 35.08 ^g^
Soy protein isolate			0.27 ± 0.07 ^b^	0.22 ± 0.01 ^fg^	1118.03 ± 11.18 ^g^	2175.93 ± 32.64 ^e^
Pea protein isolate			0.21 ± 0.04 ^b^	0.20 ± 0.01 ^h^	1379.06 ± 13.79 ^i^	2175.52 ± 32.63 ^r^
Chia protein			0.26 ± 0.03 ^b^	0.36 ± 0.00 ^d^	1512.88 ± 15.13 ^j^	1640.65 ± 24.61 ^a^
Whey protein	Sunflower Oil (SO)		0.22 ± 0.01 ^bcd^	0.22 ± 0.00 ^fg^	1349.25 ± 13.49 ^i^	2573.71 ± 38.61 ^i^
Whey protein isolate			0.20 ± 0.06 ^bcd^	0.26 ± 0.01 ^e^	487.70 ± 4.88 ^a^	2729.48 ± 40.94 ^j^
Soy protein isolate			0.25 ± 0.06 ^bcd^	0.25 ± 0.00 ^e^	516.43 ± 5.16 ^b^	2542.78 ± 38.14 ^hi^
Pea protein isolate			0.19 ± 0.03 ^bcd^	0.25 ± 0.01 ^e^	602.01 ± 6.03 ^c^	2285.05 ± 34.28 ^f^
Chia protein			0.27 ± 0.02 ^b^	0.33 ± 0.01 ^d^	1672.28 ± 16.72 ^k^	2071.40 ± 31.07 ^d^
Whey protein	Hemp Oil (HO)	Vacuum drying (VD)	0.23 ± 0.01 ^bcd^	0.49 ± 0.00 ^b^	1731.34 ± 17.31 ^l^	1952.18 ± 29.19 ^c^
Whey protein isolate			0.63 ± 0.05 ^a^	0.65 ± 0.02 ^a^	1171.59 ± 11.72 ^h^	2503.79 ± 37.56 ^h^
Pea protein isolate			0.25 ± 0.01 ^b^	0.31 ± 0.01 ^c^	1020.75 ± 10.21 ^f^	1630.44 ± 24.65 ^a^
Whey protein	Sunflower Oil (SO)		0.13 ± 0.02 ^d^	0.22 ± 0.00 ^fg^	1202.61 ± 12.04 ^h^	1946.03 ± 29.91 ^c^
Whey protein isolate			0.16 ± 0.08 ^bcd^	0.30 ± 0.01 ^d^	775.79 ± 77.65 ^d^	2385.39 ± 35.87 ^g^
Pea protein isolate			0.23 ± 0.09 ^bc^	0.24 ± 0.00 ^e^	846.88 ± 84.70 ^de^	1728.81 ± 25.39 ^b^
Sample						
Callendula Officialis (flowers)			0.33 ± 0.01 ^B^	0.30 ± 0.02 ^B^	185.26 ± 2.77 ^B^	2227.11 ± 33.40 ^B^
Isolated compound from Callendula Officialis			82.02 ± 0.57 ^A^	47.74 ± 1.06 ^A^	11.80 ± 0.18 ^A^	39.15 ± 0.78 ^A^

The data shown are mean values ± SD (n = 3), and different letters (^a,b…^—for nanoemulsion samples and ^A,B^—for extracts) in the column mean statistically significant differences, based on Tukey’s test at *p* < 0.05.

## Data Availability

Data will be made available on request.
